# Rs10204525 Polymorphism of the Programmed Death (PD-1) Gene Is Associated with Increased Risk in a Saudi Arabian Population with Colorectal Cancer

**DOI:** 10.3390/medicina58101439

**Published:** 2022-10-13

**Authors:** Nouf Al-Harbi, Mansoor-Ali Vaali-Mohammed, Suliman Al-Omar, Ahmed Zubaidi, Omar Al-Obeed, Maha-Hamadien Abdulla, Lamjed Mansour

**Affiliations:** 1 Department of Zoology, College of Science, King Saud University, Building 05, Riyadh 11451, Saudi Arabia; 2 Department of Surgery, College of Medicine, King Saud University, Riyadh 11472, Saudi Arabia

**Keywords:** colorectal cancer, immune checkpoint molecules, PD-1, SNP, genetic association, Middle East

## Abstract

Checkpoint programmed death-1 (PD-1) has been identified as an immunosuppressive molecule implicated in the immune evasion of transformed cells. It is highly expressed in tumor cells in order to evade host immunosurveillance. In this study, we aimed to assess the association between single nucleotide polymorphisms (SNP) of *PD-1* and the risk of colorectal cancer (CRC) in the Saudi population. For this case-control study, the TaqMan assay method was used for genotyping three SNPs in the *PD-1* gene in 100 CRC patients and 100 healthy controls. Associations were estimated using odds ratios (ORs) and 95% confidence intervals (95% CIs) for multiple inheritance models (codominant, dominant, recessive, over-dominant, and log-additive). Moreover, *PD-1* gene expression levels were evaluated using quantitative real-time PCR in colon cancer tissue and adjacent colon tissues. We found that the *PD-1* rs10204525 A allele was associated with an increased risk of developing CRC (OR = 2.35; *p* = 0.00657). In addition, the *PD-1* rs10204525 AA homozygote genotype was associated with a high risk of developing CRC in the codominant (OR = 21.65; *p* = 0.0014), recessive (OR = 10.97; *p* = 0.0015), and additive (OR = 1.98; *p* = 0.012) models. A weak protective effect was found for the rs2227981 GG genotype (OR = 2.52; *p* = 0.034), and no significant association was found between the rs2227982 and CRC. Haplotype analysis showed that the rs10204525, rs2227981, rs2227982 A-A-G haplotype was associated with a significantly increased risk of CRC (OR = 6.79; *p* =0.031).

## 1. Introduction

Colorectal cancer (CRC) is a malignant neoplasm that arises from the lining of the large intestine. It appears following the accumulation of multiple genetic and epigenetic alterations in cells, leading to many abnormalities in several central signaling pathways [1,2]. Both exogenous and endogenous factors contribute to the increased risk of CRC. Environmental factors including a low fiber diet, low physical activity, obesity, smoking, and alcohol consumption are all considered to be risk factors for CRC [3,4,5]. Moreover, inherited genetic factors have been shown to contribute significantly to the susceptibility of CRC, as for many other cancer diseases [6,7,8,9]. In addition, the risk of CRC is higher in individuals with inflammatory bowel diseases such as Crohn’s disease, which involve alterations in immune responses [10].

Genome-wide association studies (GWASs) have improved the search for genetic variations such as single nucleotide polymorphisms (SNPs) which are related to many diseases including cancer. A significant number of SNPs have been reported as a part of disease susceptibility to CRC. In the European population, GWASs have reported almost 100 SNPs associated with CRC risk, mapped in more than 50 risk loci [6,11,12]. In addition, in Asian populations, 18 SNPs associated with CRC mapped in 16 risk loci have been reported, and some of them overlap with European loci [13,14]. Among these SNPs, a large number were related to immune system functioning [15,16]. Studies on the implication of SNPs in genes coding for functional immune molecules, especially checkpoint molecules, have shown a strong relationship with the efficiency of the immune system in fighting cancer cells [17,18].

Checkpoint programmed death-1 (PD-1)/programmed cell death ligands (PD-Ls) have been identified as immunosuppressive molecules implicated in the immune evasion of transformed cells. PD-1 (also known as PDCD1) is a type I transmembrane glycoprotein which belongs to the CD28 family and is a member of the B7-CD28 superfamily. The *PD-1* gene is located on chromosome 2q37.3 and functions as an immune-inhibitory receptor. Ligands of PD-1 include PD-L1 (B7-H1) and PD-L2 (B7-DC). *PD-1* is expressed on activated T cells, B cells, NKT cells, and monocytes [19]. Programmed death-1-ligand 1 (PD-L1) is highly expressed by tumor cells as an adaptive mechanism to evade host immunosurveillance. *PD-1* overexpression results in the exhaustion of CD8+ leading to a reduced antitumor immune response and hence tumor progression. Variations such as SNPs could influence the gene at the transcriptional level. Several types of cancers and immune diseases have been linked to genetic polymorphisms in the *PD-1* and *PD-L1* genes [20,21,22,23].

Several studies have indicated that the upregulation of *PD-1* and its ligands results in an inhibition of immune responses represented by the exhaustion of T cells during chronic viral disease [24,25,26]. Other studies have reported that polymorphisms in the *PD-1* gene contribute to the progression of several autoimmune diseases, infectious diseases, and cancer [27,28,29,30]. Among the several *PD-1* SNPs, few polymorphisms have been investigated for their relationships with cancer and immune disease. These include the *PD-1.5* (rs2227981), *PD-1.6* (rs10204525), and *PD-1.9* (rs2227982) polymorphisms which were correlated with lung adenocarcinoma [31], cervical [32], lung [33], gastric [34], and thyroid cancers [35], and esophageal cancer [26].

Few studies have investigated the role and correlation between SNPs in the *PD-1* gene and CRC pathogenesis, and the results have been controversial [2,36,37]. To the best of our knowledge, no studies have reported on the association between *PD-1* gene polymorphism and immune or cancerous diseases across the countries of the Arabian Peninsula. Hence, the purpose of this study was to examine the association between three functional SNPs in the *PD-1* gene and colorectal cancer in Saudi Arabia.

## 2. Materials and Methods

### 2.1. Study Population

A total of 200 blood samples were obtained from King Khalid University Hospital. These encompassed 100 patients who were diagnosed with sporadic colorectal cancer at the Department of Colorectal Cancer and 100 healthy controls with no history of cancer. The group of CRC patients included individuals with a mean age 56.14 and 65% males (Table 1). Patient blood samples were collected preoperatively between 2014 and 2016. None of the patients underwent preoperative radiation or chemotherapy treatment. Patients and controls were of Saudi Arabian ethnicity. All controls were gender- and age-matched and recruited from physical examinations after the diagnostic exclusion of personnel or those with a familial history of cancer and other autoimmune or chronic diseases. The study complied with ethical requirements and has been approved by the Ethics Committee of King Saud University. Patient consent was obtained for this study.

### 2.2. DNA Extraction

Blood samples of approximately 3 mL were collected in vacutainers containing ethylenediaminetetraacetic acid (EDTA) from all subjects enrolled in the study. Genomic DNA was extracted from peripheral blood using the QIAamp DNA Blood Mini Kit (Qiagen, Valencia, CA, USA). The DNA was quantitated spectrophotometrically on a NanoDrop 8000 (Thermo Scientific, Waltham, MA, USA), and its purity was examined using standard A260/A280 and A260/A230 ratios, then stored at −80 °C until use.

### 2.3. SNP Selection and Genotyping

For this genetic association study, three *PD-1* SNPs were selected; rs10204525 (*PD-1.6*), rs2227981 (*PD-1.5*), and rs2227982 (*PD-1.9*). SNPs were acquired from Thermo Fisher Scientific (Thermo Fisher Scientific, Waltham, MA, USA). The characteristics of each SNP are presented in Table 2. Genotyping was performed using a TaqMan allelic discrimination assay as previously described [38]. Genotyping were performed according to the manufacturer’s protocol. Briefly, for each PCR reaction, a 20-ng DNA sample was used with 10 μL of 2X Universal Master Mix and 1X assay mix in a total reaction volume of 20 μL (Applied Biosystems, Foster City, CA, USA). PCR conditions were as follows: pre-read stage at 60 °C for 30 s, hold stage at 95 °C for 10 min, PCR stage at 95 °C for 15 s and 60 °C for 1 min for 40 cycles, and post-read stage at 60 °C for 30 s. All genotypes were determined using end-point reading on a ViiATM 7 Real-Time PCR System (Applied Biosystems, Foster City, CA, USA). For quality control, 5% of samples were randomly selected and subjected to repeat analysis as a measure of verification for the genotyping procedures. The results were reproducible without any discrepancies. For each DNA sample, allelic genotyping was used for the detection of three SNPs in the *PD-1* gene.

### 2.4. RNA Extraction and Real-Time Quantitative Polymerase Chain Reaction

RNA was extracted from tumor and healthy tissues using the PARIS™ kit (Ambion, Foster City, CA, USA). A high-capacity cDNA kit was used for reverse transcription (cat. no. 4368814; Applied Biosystems, Foster City, CA, USA). The quality of RNA was evaluated by assessing the A260/280 ratio (1.8–2.0).

Quantitative PCR analysis was performed on a ViiA™ 7 Real-Time PCR System (Thermo Fisher Scientific) using SYBR Green PCR Master Mix (Thermo Fisher Scientific).

Relative quantification (RQ) was calculated using the comparative CT method (2(−∆∆Ct)) [38]. Normal tissue samples were used as a calibrator, and GAPDH as a reference gene for normalization. The data were expressed as median values.

### 2.5. In Silico Analysis

In silico screening for identifying microRNA binding sites in the 3′ UTR of PD-1, encompassing the region rs10204525, was performed using miRNASNiPer (http://vm24141.virt.gwdg.de/services/microsniper, accessed on 4 October 2022) [39] and miRNASNP-v3 [40]. Functional characterization of the miR-seed polymorphisms was performed with TargetScan (http://www.targetscan.org/, accessed on 4 October 2022), used to analyze whether the miR-seed-SNP caused the formation of seed regions for the identified miRNA [41], and the RNAcofold web server to predict secondary structures and estimate the minimum free energy (MFE) [42]. Finally, the predictSNP platform (https://loschmidt.chemi.muni.cz/predictsnp2, accessed on 4 October 2022) was used to predict the deleterious effect of SNPs based on the evaluation of six tools for variant prioritization: CADD, DANN, FATHMM, FitCons, FunSeq2, and GWAVA [43].

### 2.6. Statistical Analysis

Association analysis including the codominant, dominant, recessive, over-dominant, and log-additive models were performed using web-based software (SNPStats) [44]. The inheritance model with the lowest Akaike information criteria (AIC) value was considered as the best fit. Odds ratios (OR) with 95% confidence intervals (CI) were calculated based on logistic regression. Haplotype analysis and linkage disequilibrium (LD) were also performed using SNPStats and Haploview software (http://www.broadinstitute.org/haploview/haploview/index.php, accessed on 29 August 2022).

Haplotype frequency was estimated through a statistical method to assess the differences in haplotype frequency distribution between cases and controls. For each SNP, deviation from a Hardy–Weinberg equilibrium and χ^2^ values were calculated using the web-based tool at https://ihg.helmholtz-muenchen.de/cgi-bin/hw/hwa1.pl, accessed on 2 September 2022.

Haldane–Anscombe correction was applied to estimate the OR for zero values. A *p*-value of <0.05 was considered as significant.

## 3. Results

### 3.1. Demographic Characteristics of Study Population

Patient baseline characteristics are shown in Table 1. The study included 200 participants, 100 patients with sporadic colorectal cancer and 100 healthy matched controls. The patient group comprised 64 males and 36 females with an average age group of 56.33 ± 14.56. The control group comprised 65 males and 35 females with an average age group of 56.31 ± 14.56 (age- and gender-matched controls). Patients were classified according to the three TNM stages: I, II, and III. In total, 57% belonged to stage II and 32% to stage III, and only 11% to stage I. For all subjects, genotyping of the *PD-1* gene was performed using a TaqMan assay for three selected SNPs; *PD-1.6*, *PD-1.5,* and *PD-1.9* (Table 2).

### 3.2. PD-1 SNP Association with CRC

The genetic and allelic association between the three SNPs and CRC was tested using five genetic models (allelic, codominant, dominant, recessive, over-dominant, and additive). The results are presented in Table 3. The distribution of genotypes for *PD-1.6* and *PD-1.5* in the control group followed a Hardy–Weinberg equilibrium (*p* > 0.05, χ^2^ < 3.84), while the *PD-1.9* (*p* = 0.016, χ^2^ = 8.22) was deviated from a Hardy–Weinberg equilibrium.

The genotyping of *PD-1.6* showed that the AA genotype was present in 9% of the patients; however, it was lacking among controls. Statistically, the AA genotype was significantly associated with an increased risk of CRC in the codominant (OR = 21.65; 95% CI (1.23–378.73); *p* = 0.0014), recessive (OR = 10.97; 95% CI (1.37–87.43); *p* = 0.0015), and additive (OR = 1.98; 95% CI (1.14–3.45); *p* = 0.012) models, after applying Haldane–Anscombe correction. The A allele was significantly high in frequency in CRC patients compared to the healthy control group (0.19 vs. 0.09), suggesting an increased susceptibility to CRC for individuals sharing this allele (OR = 2.35; 95% CI (1.24–4.03); *p* = 0.00657).

For the *PD-1.5* polymorphism, our analysis showed that the AA genotype was the most frequent in both patients and controls, followed by the AG and GG genotypes, respectively. In the codominant model, the GG genotype was found with a low frequency in CRC (8%) compared to healthy controls (18%), although this difference did not reach significance (OR = 2.25; 95% CI (0.90–5.63); *p* = 0.078). However, in the recessive model, a positive correlation with disease was observed for AG + AA vs. G/G (OR = 2.52; 95% CI (1.04–6.11); *p* = 0.034) with the lowest AIC (276). Furthermore, the A allele was the most common allele in comparison to the G allele, which does not coincide with the MAF database (A = 0.33/72 (Qatari), A = 0.35/1759 (1000Genomes) (Table 1). The dominant genotype AA against the AG + GG genotype had a frequency of 52 to 48 with no differences between patients and controls.

Our analysis for *PD-1.9* did not show any significant association with CRC in any of the studied models.

### 3.3. Age and Gender Stratified Analysis

In order to investigate the possible influence of the SNPs according to age and gender, we have stratified the 100 cases into two subgroups for each parameter. For age-stratified analysis, we classified the patients to those aged ≥56 (*n* = 59) and those aged <56 years (*n* = 41). For gender stratification, the group of females comprised 36 individuals versus 64 males. The allelic and genetic distribution of the three SNPs among each group was evaluated using the five models of inheritance. Our analysis revealed no significant associations between the three SNPs in relation to age (Table 4) or gender (Table 5).

### 3.4. Haplotype Analysis

**Figure 1:**
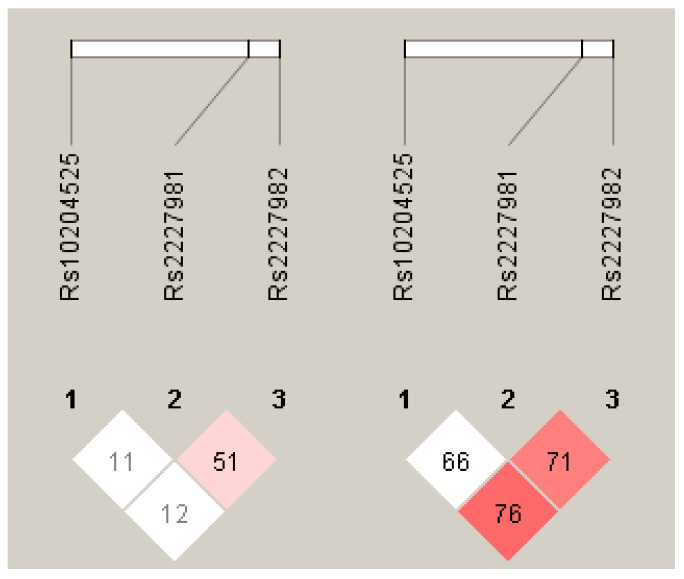
Linkage disequilibrium (LD) plots of three SNPs in the *PD-1* gene region. The plot for subjects with CRC (**left**) and control (**right**) were generated by Haploview. The numbers in the squares show the D’ values between the SNPs, expressed as percentages within the respective squares.

Linkage disequilibrium (LD) analysis in the control samples revealed a weak LD between *PD-1.5* and *PD-1.9,* and no LD for *PD-1.6* (Figure 1). The haplotypes were generated using the three SNPs among cases and controls (Table 6). Six common haplotypes of *PD-1.6, PD-1.5*, and *PD-1.9* (frequency > 1.8%) showed an accumulated frequency of more than 95% of haplotypes. The distribution of haplotypes exhibited differences between cases and controls. The most frequent haplotype was the G-A-G, with 28% in CRC cases and 41% in healthy controls, which was used as a reference. The haplotype A-A-G was associated with an increased risk of CRC (OR = 6.79; 95% CI (1.21–38.25); *p* = 0.031). Globally, an association between haplotypes and diseases was supported by the global *p*-value (*p*-value = 0.017) (Table 6).

### 3.5. Gene Expression Analysis of PD-1 mRNA

**Figure 2:**
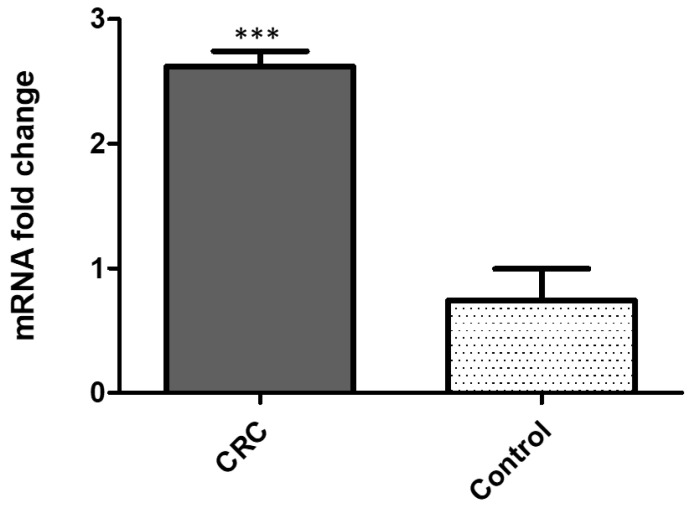
*PDCD1* gene expression assessed by qRT-PCR in colon cancer tissues compared to normal matching tissues: (mean ± SD, data normalized to GAPDH, *** *p* < 0.00 *t*-test).

The quantification of *PD-1* gene expression from 35 colon cancer fresh tissues and 35 normal adjacent matching tissues was performed using real-time reverse transcriptase PCR (qRT-PCR). The expression of *PD-1* mRNA was significantly higher (4–8-fold difference; *p* < 0.001) in colon cancer tissues as compared to healthy adjacent colon samples (Figure 2).

### 3.6. In Silico Functional Analysis of the PD-1.6 Polymorphism

**Figure 3:**
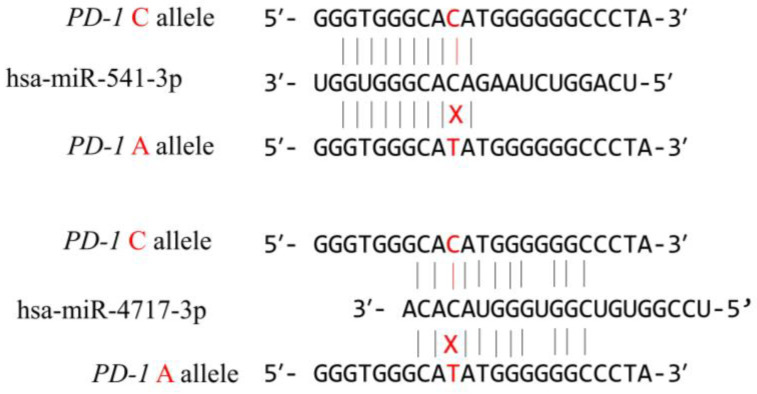
Putative miRNA binding sites in wild type and mutant allele, showing the disruption of the interaction between miRNA and its predicted target site in mutant type.

In order to evaluate the functional effect of the rs10204525 polymorphism located in the 3′ UTR of the *PD-1* gene, we have performed in silico analysis. Using the MicroSNiPer prediction website, we have identified the three most plausible miRNAs which possess a 8–10-nt seed length and recognize the miRNA response element (MRE), enclosing the *PD-1.6* site (Table 7). The MFE and ΔMFE are the lowest for hsa-miR-541-3p and hsa-miR-4717-3p, and thus these could be considered as potential miRNAs that regulate gene expression through the MRE that includes the *PD-1.6* polymorphism (Figure 3). A genome-wide annotation of variant analysis (GWAVA) predicted a deleterious effect of the *PD-1.6* mutation with 78% accuracy.

## 4. Discussion

Functional polymorphisms in immune checkpoint genes are reported to have a serious impact on the outcomes of many types of cancers [17,45]. In fact, there is an increasing focus on the role of SNPs in the modulation of individual susceptibility or protection against cancer. Large-scale GWASs have identified many loci associated with the risk of CRC [6,7,8,9,46]. In this context, certain polymorphisms in the *PD-1* gene have been studied and found to contribute to the individual risk of cancer [28,47,48]. Studies have also reported changes in the expression of *PD-1* and an association with many types of cancers [21,49,50,51]. In the current study, we investigated the relationship between the *PD-1*.6 (rs10204525), *PD-1*.5 (rs2227981), and *PD-1*.9 (rs2227982) polymorphisms and the risk of developing CRC in Saudi Arabia. In our study, a strong association between the A allele and AA genotype of *PD-1*.6 and CRC was found in almost all tested inheritance models. For the two other *PD-1* polymorphisms (*PD-1*.5 and *PD-1*.9), an association with the disease was found only for the GG genotype of *PD-1*.5 in the recessive model. For the *PD-1* gene, few studies have reported an association between the SNPs tested herein and cancer diseases. The *PD-1.6* polymorphism is reported to be associated with an increased susceptibility of some clinical features of esophageal cancer in the Chinese Han population [52]. Additionally, this SNP was correlated with the susceptibility to human T-cell leukemia virus type 1 and some clinical features in an Iranian population [53]. However, in a recent study reported by Fathi, et al. [54], no association between *PD-1.6* and basal cell carcinoma (BCC) was found. Similarly, in two other separate case-control studies, no associations were detected between *PD-1.6* polymorphism and the risk of developing CRC in subjects from Heilongjiang Province in Northeast China [2] and Eastern China [36]. However, associations between *PD-1.6* polymorphisms and several infectious and autoimmune diseases have been reported in many studies [20,27,29,30,32,55,56]. For *PD-1.5* and *PD-1.9*, controversial results have been reported for their association with cancer diseases. Two separate studies reported no association between *PD-1.9* and CRC in two populations in China, conducted by Ge et al. [2] and Lin et al. [36]. However, an association between *PD-1.5* and an increased susceptibility to colon cancer and rectal cancer was reported earlier by Mojtahedi et al. [37] in an Iranian population. In a study reported by Zhao et al. [22], who examined the association of the three SNPs (*PD-1*.6, *PD-1*.5, and *PD-1*.9) with CRC among a Han Chinese population, no significant connection was found with the disease. In other case-control studies regarding the relationship between *PD-1.5* and *PD-1.9* and other cancer diseases, including lung adenocarcinoma [31], cervical cancer [32], breast cancer [28], gastric cancer [34], and thyroid cancer [35], significant associations have been reported. This discrepancy in results could be explained by ethnic factors alongside other environmental factors that are usually not considered in the association analysis, such as smoking, diet, alcohol drinking, ultraviolet and ionizing radiation exposition, medical procedures and drugs, occupation, reproductive behavior, pollution, infection, and other still unknown factors [3,4,5]. These environmental factors could act by inducing somatic mutations or epigenetic effects that could modify the structure of DNA or affect its expression or stability [57,58]. In this study, we tested the relationships between the *PD-1.6*, *PD-1.5,* and *PD-1.9* haplotypes and CRC, and we found a strong positive association between the A-A-G haplotype and an increased risk of CRC. In a study reported by Zhao et al. [22], a protective effect of the A-G-A of the same SNPs against CRC was found among the Han Chinese population. This haplotype was found with a very low frequency in our population. Moreover, we have explored the gene expression levels in colon cancer tissues in comparison to surrounding normal colon tissues. Our results showed an up-regulation of the *PD-1* gene in colorectal tumor cells. This result is in agreement with other studies on the association of high-level *PD-1* expression with the clinical features of multiple tumors including CRC, hepatocellular carcinoma, esophageal cancer, and kidney clear cell carcinoma [21,26,49,50,51,59]. Thus, immunotherapy based on monoclonal antibodies against PD-1 proteins, blocking the binding with PD-L1, have demonstrated the efficacy against some malignant diseases [60,61]. Moreover, the *PD-1.6* polymorphism has been associated with an overexpression of the *PD-1* gene [59]. It has been shown that this polymorphism is involved in the activation and transcription of *PD-1*. In our in silico analysis, we have identified three putative and allele-sensitive miRNA molecules that could interact with the region enclosing the *PD-1.6* polymorphism. miR-541 and miR-4717 were found to be the most plausible; they could play important roles in the regulation of *PD-1* gene expression and are sensitive to *PD-1.6* polymorphism. In an experimental study, Zhang, et al. [62] showed that miR-4717 may interact in an allele-specific manner with the 3′ UTR of *PD-1.6* and regulate *PD-1* expression. Therefore, lymphocytes from patients with chronic HBV harboring the *PD-1.6* GG genotype and transfected with miR-4717 mimics exhibited significantly decreased *PD-1* expression and increased TNF-α and IFN-γ production, although no effect was observed with the *PD-1.6* AA genotype carriers [62]. Further studies are necessary to investigate the role of *PD-1.6* polymorphism and its association with *PD-1* expression for eventual use in specific tailored immunotherapy against cancer diseases.

## 5. Conclusions

In conclusion, our findings support an association between the *PD-1* rs10204525 polymorphism and an increased CRC risk in the Saudi population. Additional reports involving larger sample sizes with more detailed clinical information in different ethnicities will be important to confirm our conclusions.

## Figures and Tables

**Table 1:** Demographic and main clinical data of CRC patients and controls used for *PD-1* SNP genotyping.

Characteristics			CRC (100)	Control (100)
Gender (number)	Male		64	65
	Female		36	35
Age (average ± SD)			56.33 ± 14.56	56.31 ± 14.56
Localization	Colon		40	-
	Recto-sigmoid		60	-
Stages		I	11	-
		II	57	-
		III	32	-
		IV	0	-

**Table 2:** Characteristics of selected polymorphisms involved in *PD-1* gene receptor 4.

SNP ID/Assay ID	Common Name	Chromosome Position	NucleotideChange	Region	MAF in Human Populations			Present Study
					Global	European	South Asian	
Rs10204525	PD-1.6	Chr2/241851121	G/A	3′ UTR	A = 0.35	A = 0.12	A = 0.19	A = 0.19
Rs2227981	PD-1.5	Chr 2/241850169	A/G	Exon 5	A = 0.35	A = 0.40	A = 0.24	A = 0.67
Rs2227982	PD-1.9	Chr 2/242793433	G/A	Exon 5	A = 0.14	A = 0.01	A = 0.06	A = 0.34

MAF: minor allele frequency.

**Table 3:** Distribution of *PD-1* SNPs genotypes and allele frequencies in colorectal cancer cases and a control population.

Locus	Model	Genotype	CRC (%) *n* = 100	Controls (%) *n* = 100	OR (95% CI)	*p*-Value	AIC
Rs10204525 G > A	Alleles	G	0.81	0.91	1		
		A	0.19	0.09	2.35 (1.24–4.03)	**0.00657**	
	Codominant	GG	71 (71%)	81 (81%)	1.00		
		GA	20 (20%)	19 (19%)	1.20 (0.59–2.43)		270.1
		AA	9 (9%)	0 (0%)	**21.65 (1.23–378.73)**	**0.0014**	
	Dominant	GG	71 (71%)	81 (81%)	1.00		
		GA + AA	29 (29%)	19 (19%)	1.74 (0.90–3.37)	0.097	278.5
	Recessive	GA + GG	91 (91%)	100 (100%)	1.00		
		AA	9 (9%)	0 (0%)	**10.97 (1.37–87.43)**	**0.0015**	268.4
	Over-Dominant	GG + AA	80 (80%)	81 (81%)	1.00		
		GA	20 (20%)	19 (19%)	1.07 (0.53–2.15)	0.86	281.2
	Log-Additive				**1.98 (1.14–3.45)**	**0.012**	274.9
Rs2227981 A > G	Alleles	A	0.67	0.72	Ref		-
		G	0.33	0.28	0.79 (0.43–1.11)	0.53	
	Codominant	AA	52 (52%)	52 (52%)	1.00	1	277.9
		AG	30 (30%)	40 (40%)	0.75 (0.41–1.38)		
		GG	18 (18%)	8 (8%)	2.25 (0.90–5.63)	0.078	
	Dominant	AA	52 (52%)	52 (52%)	1.00		
		AG + GG	48 (48%)	48 (48%)	1.00 (0.57–1.74)	1	281.3
	Recessive	AG + AA	82 (82%)	92 (92%)	1.00		
		GG	18 (18%)	8 (8%)	**2.52 (1.04–6.11)**	**0.034**	276.7
	Over-Dominant	AA-GG	70 (70%)	60 (60%)	1.00	0.14	279.1
		AG	30 (30%)	40 (40%)	0.64 (0.36–1.15)		
	Log-Additive				1.22 (0.82–1.82)	0.32	280.3
Rs2227982 G > A	Alleles	G	0.66	0.68	1		
		A	0.34	0.32	0.93 (0.61–1.41)	0.83	
	Codominant	GG	38 (38%)	40 (40%)	1.00		
		AG	57 (57%)	56 (56%)	1.07 (0.60–1.91)	0.92	283.1
		AA	5 (5%)	4 (4%)	1.32 (0.33–5.27)		
	Dominant	GG	38 (38%)	40 (40%)	1.00		
		AG+ AA	62 (62%)	60 (60%)	1.09 (0.62–1.92)	0.77	281.2
	Recessive	GG+ AG	95 (95%)	96 (96%)	1.00		
		AA	5 (5%)	4 (4%)	1.26 (0.33–4.85)	0.73	281.1
	Over-Dominant	AA +GG	43 (43%)	44 (44%)	1.00		
		AG	57 (57%)	56 (56%)	1.04 (0.60–1.82)	0.89	281.2
	Log-Additive		---	---	1.10 (0.67–1.80)	0.71	

CRC: colorectal cancer, OR: odds ratio, 95% CI: 95% confidence interval, *p* < 0.05 was considered significant and these results are depicted in bold.

**Table 4:** Association of *PD-1* SNP polymorphisms with CRC after age stratification.

Locus	Model	Genotype	CRC <56 (%) *n* = 41	CRC ≥56 (%) *n* = 59	OR (95% CI)	*p*-Value	AIC
Rs10204525	Allele	G	0.77	0.87	1		
		A	0.23	0.13	0.52 (0.24–1.24)	0.09	
	Codominant	GG	31 (75.6%)	40 (67.8%)	1	0.12	137.1
		GA	9 (21.9%)	11 (18.6%)	0.95 (0.35–2.57)		
		AA	1 (2.4%)	8 (13.6%)	6.20 (0.74–52.23)		
	Dominant	GG	31 (75.6%)	40 (67.8%)	1	0.39	138.6
		GA-AA	10 (24.4%)	19 (32.2%)	1.47 (0.60–3.61)		
	Recessive	GG-GA	40 (97.6%)	51 (86.4%)	1	0.039	135.1
		AA	1 (2.4%)	8 (13.6%)	6.27 (0.75–52.26)		
	Over-Dominant	GG-AA	32 (78%)	48 (81.4%)	1	0.69	139.2
		GA	9 (21.9%)	11 (18.6%)	0.81 (0.30–2.19)		
	Log-Additive	---	---	---	1.63 (0.83–3.20)	0.14	137.2
Rs2227981 A/G	Allele	A	0.68	0.66	Ref		-
		G	0.32	0.34	0.9 (0.49–1.65)	0.74	
	Codominant	AA	22 (53.7%)	30 (50.9%)	1	0.96	141.3
		AG	12 (29.3%)	18 (30.5%)	1.10 (0.44–2.74)		
		GG	7 (17.1%)	11 (18.6%)	1.15 (0.39–3.45)		
	Dominant	AA	22 (53.7%)	30 (50.9%)	1	0.78	139.3
		AG-GG	19 (46.3%)	29 (49.1%)	1.12 (0.50–2.49)		
	Recessive	AA-AG	34 (82.9%)	48 (81.4%)	1	0.84	139.3
		GG	7 (17.1%)	11 (18.6%)	1.11 (0.39–3.16)		
	Over-Dominant	AA-GG	29 (70.7%)	41 (69.5%)	1	0.89	139.4
		A/G	12 (29.3%)	18 (30.5%)	1.06 (0.44–2.54)		
	Log-Additive	---	---	---	1.08 (0.64–1.82)	0.78	139.3
Rs2227982	Allele	G	0.71	0.64			
		A	0.29	0.36	0.77 (0.39–1.32)	0.29	
	Codominant	GG	19 (46.3%)	19 (32.2%)	1	0.35	139.3
		AG	20 (48.8%)	37 (62.7%)	1.85 (0.80–4.27)		
		AA	2 (4.9%)	3 (5.1%)	1.50 (0.22–10.02)		
	Dominant	GG	19 (46.3%)	19 (32.2%)	1		
		AG-AA	22 (53.7%)	40 (67.8%)	1.82 (0.80–4.13)	0.15	137.3
	Recessive	GG-AG	39 (95.1%)	56 (94.9%)	1		
		AA	2 (4.9%)	3 (5.1%)	1.04 (0.17–6.55)	0.96	139.4
	Over-Dominant	GG-AA	21 (51.2%)	22 (37.3%)	1		
		AG	20 (48.8%)	37 (62.7%)	1.77 (0.79–3.96)	0.17	137.5
	Log-Additive	---	---	---	1.57 (0.77–3.23)	0.21	137.8

**Table 5:** Association of *PD-1* SNP polymorphisms with CRC after gender stratification.

Locus	Model	Genotype	CRC Female *n* = 36	CRC Male *n* = 64	OR (95% CI)	*p*-Value	AIC
Rs10204525	Allele	G	0.82	0.8	1		
		A	0.18	0.2	1.102 (0.524–2.315)	0.79	
	Codominant	GG	25 (69.4%)	46 (71.9%)	1		
		GA	9 (25%)	11 (17.2%)	0.66 (0.24–1.82)	0.47	135.2
		AA	2 (5.6%)	7 (10.9%)	1.90 (0.37–9.86)		
	Dominant	GG	25 (69.4%)	46 (71.9%)	1		
		GA-AA	11 (30.6%)	18 (28.1%)	0.89(0.36–2.18)	0.8	134.6
	Recessive	GG-GA	34 (94.4%)	57 (89%)	1		
		AA	2 (5.6%)	7 (10.9%)	2.09 (0.41–10.63)	0.35	133.8
	Over-Dominant	GG-AA	27 (75%)	53 (82.8%)	1		
		GA	9 (25%)	11 (17.2%)	0.62 (0.23–1.68)	0.35	133.8
	Log-Additive	---	---	---	1.07 (0.57–2.04)	0.83	134.6
Rs2227981 A/G	Allele	A	0.6	0.71	Ref		-
		G	0.4	0.29	0.603 (0.329–1.106)	0.10	
	Codominant	AA	16 (44.4%)	36 (56.2%)	1	0.35	134.6
		AG	11 (30.6%)	19 (29.7%)	0.77 (0.30–1.98)		
		GG	9 (25%)	9 (14.1%)	0.44 (0.15–1.33)		
	Dominant	AA	16 (44.4%)	36 (56.2%)	1	0.26	133.4
		AG-GG	20 (55.6%)	28 (43.8%)	0.62 (0.27–1.42)		
	Recessive	AA-AG	27 (75%)	55 (85.9%)	1	0.18	132.9
		GG	9 (25%)	9 (14.1%)	0.49 (0.17–1.38)		
	Over-Dominant	AA-GG	25 (69.4%)	45 (70.3%)	1	0.93	134.7
		AG	11 (30.6%)	19 (29.7%)	0.96 (0.39–2.33)		
	Log-Additive	---	---	---	0.68 (0.40–1.16)	0.16	132.7
Rs2227982	Allele	G	0.67	0.66	Ref		
		A	0.33	0.34	1.012 (0.549–1.866)	0.970	
	Codominant	GG	13(36.1%)	25 (39.1%)	1		
		AG	22 (61.1%)	35 (54.7%)	0.83 (0.35–1.95)	0.66	135.9
		AA	1 (2.8%)	4 (6.2%)	2.08 (0.21–20.57)		
	Dominant	GG	13(36.1%)	25 (39.1%)	1		
		AG-AA	23 (63.9%)	39(60.9%)	0.88 (0.38–2.05)	0.77	134.6
	Recessive	GG-AG	35 (97.2%)	60 (93.8%)	1		
		AA	1 (2.8%)	4 (6.2%)	2.33 (0.25–21.71)	0.42	134
	Over-Dominant	GG-AA	14 (38.9%)	29 (45.3%)	1		
		AG	22 (61.1%)	35 (54.7%)	0.77 (0.33–1.76)	0.53	134.3
	Log-Additive	---	---	---	1.02 (0.49–2.09)	0.96	134.7

**Table 6:** Association of *PD-1* haplotypes with CRC in Saudi Arabia.

Rs10204525	Rs2227982	Rs2227981	CRC	Control	OR (95% CI)	*p*-Value
G	A	G	0.28	0.41	Ref	
G	A	A	0.24	0.23	1.27 (0.66–2.45)	0.47
G	G	G	0.25	0.26	1.73 (0.73–4.10)	0.21
A	A	G	0.11	0.02	**6.79 (1.21–38.25)**	**0.031**
A	A	A	0.03	0.07	0.56 (0.12–2.67)	0.46
G	G	A	0.04	0.02	2.66 (0.70–10.04)	0.15

Significant values(*p* < 0.05) are highlighted in bold.

**Table 7:** Putative miRNA targeting *PD-1.6* SNPs in the 3′ UTR of the *PD-1* gene, and the difference in the free energy of hybridization (ΔFME) for wild-type and variant alleles.

			MFE (kcal/mol)		
Putative miRNAs	Length	Seed Region	Wild Allele	Mutant Allele	ΔMFE (kcal/mol)
hsa-miR-541-3p	22	10	−26.1	−24.4	−1.7
hsa-miR-4717-3p	22	8	−17.00	−15.68	−1.33
hsa-miR-5189	24	8	−12.25	−11.97	−28

## Data Availability

All data relevant to the study are included in the article.
